# Effect of a Consumer-Focused Website for Low Back Pain on Health Literacy, Treatment Choices, and Clinical Outcomes: Randomized Controlled Trial

**DOI:** 10.2196/27860

**Published:** 2021-06-15

**Authors:** Paul William Hodges, Leanne Hall, Jenny Setchell, Simon French, Jessica Kasza, Kim Bennell, David Hunter, Bill Vicenzino, Samuel Crofts, Chris Dickson, Manuela Ferreira

**Affiliations:** 1 School of Health and Rehabilitation Sciences The University of Queensland Brisbane Australia; 2 Department of Chiropractic Macquarie University Sydney Australia; 3 School of Public Health and Preventive Medicine Monash University Melbourne Australia; 4 Centre for Health Exercise and Sports Medicine Department of Physiotherapy University of Melbourne Melbourne Australia; 5 Institute of Bone and Joint Research, The Kolling Institute Northern Clinical School, Faculty of Medicine and Health University of Sydney Sydney Australia; 6 Centre for Epidemiology and Biostatistics The University of Melbourne Melbourne Australia; 7 Department of Integrative Medicine Chris O'Brien Lifehouse Hospital Sydney Australia

**Keywords:** low back pain, randomized controlled trial, internet resources, health literacy

## Abstract

**Background:**

The internet is used for information related to health conditions, including low back pain (LBP), but most LBP websites provide inaccurate information. Few studies have investigated the effectiveness of internet resources in changing health literacy or treatment choices.

**Objective:**

This study aims to evaluate the effectiveness of the MyBackPain website compared with unguided internet use on health literacy, choice of treatments, and clinical outcomes in people with LBP.

**Methods:**

This was a pragmatic, web-based, participant- and assessor-blinded randomized trial of individuals with LBP stratified by duration. Participants were randomly allocated to have access to the evidence-based MyBackPain website, which was designed with input from consumers and expert consensus or unguided internet use. The coprimary outcomes were two dimensions of the Health Literacy Questionnaire (dimension 2: “having sufficient information to manage my health;” dimension 3: “actively managing my health;” converted to scores 1-100) at 3 months. Secondary outcomes included additional Health Literacy Questionnaire dimensions, quality of treatment choices, and clinical outcomes.

**Results:**

A total of 453 participants were recruited, and 321 (70.9%) completed the primary outcomes. Access to MyBackPain was not superior to unguided internet use on primary outcomes (dimension 2: mean difference −0.87 units, 95% CI −3.56 to 1.82; dimension 3: mean difference −0.41 units, 95% CI −2.78 to 1.96). Between-group differences in other secondary outcomes had inconsistent directions and were unlikely to be clinically important, although a small improvement of unclear importance in the quality of stated treatment choices at 1 month was found (mean difference 0.93 units, 95% CI 0.03 to 1.84).

**Conclusions:**

MyBackPain was not superior to unguided internet use for health literacy, but data suggest some short-term improvement in treatment choices. Future research should investigate if greater interactivity and engagement with the website may enhance its impact.

**Trial Registration:**

Australian New Zealand Clinical Trials Registry (ANZCTR) ACTRN12617001292369; https://www.anzctr.org.au/Trial/Registration/TrialReview.aspx?id=372926

**International Registered Report Identifier (IRRID):**

RR2-10.1136/bmjopen-2018-027516

## Introduction

### Background

Low back pain (LBP) is the leading cause of disability globally [1]. Unnecessary and ineffective management inflates its burden [2], and its impact is worsened by negative messages and beliefs [3,4]. Access to an evidence-based, consumer-focused tool to enhance health literacy and empower active participation in self-management and treatment selection could reduce the burden. Although tools are available, the evaluation of their effectiveness is limited.

The internet is a primary source of health-related information [5-7]. Up to 68% of individuals search for health-related information on the web [7,8]. Information is sought on the internet for treatment decision support [7,9,10], self-management advice [7,9], guidance regarding health care providers [10], increased knowledge [10,11], and preparation for consultations [10]. The advantages of the internet are accessibility, high reach, low cost, and scalability [12]. Unfortunately, most LBP websites are rated poorly [13-15], do not meet consumers’ needs [16], provide inaccurate information and treatment recommendations [17], and use inappropriate language [14]. Although systematic reviews provide modest evidence of the efficacy of internet-delivered *interventions* (eg, cognitive behavioral therapy [18,19]), whether *information* resources improve outcomes and behaviors is unknown.

### Objectives

This study aims to evaluate access to an LBP website [20] that integrates evidence-based LBP information developed through consumer consultation and expert consensus [21]. We hypothesize that the MyBackPain website more effectively improves health literacy (primary outcome), choice of evidence-based treatments, and clinical outcomes in people with LBP than unguided use of internet resources.

## Methods

### Trial Design

We conducted a two-arm pragmatic, web-based, superiority randomized controlled trial. It was prospectively registered (Australian New Zealand Clinical Trials Registry ACTRN12617001292369), and the protocol has been published [22]. Ethical approval was obtained from the University of Queensland Ethics Committee (#2017000995). Written informed consent was obtained from all participants.

### Participants

We recruited community participants with current LBP using newsletters, email lists, consumer groups, websites, social media, and newsletters to members of a health insurer (Medibank Private). Inclusion criteria were current LBP of any duration, aged 18 years and above (no upper age limit), current residence in Australia, adequate English to use the MyBackPain website, and internet access. Participants were excluded if they reported a previous or existing serious spinal pathology (fracture, cancer, or infection) or specific diagnosis including sciatica (participants with leg pain but no diagnosis of sciatica were not excluded), lumbar spinal stenosis, or nerve root compromise.

### Procedure

This trial was conducted on the web. Potential participants were provided with a web link to the participant information sheet and consent form. Those who consented completed an eligibility screening form and, if eligible, were invited to complete the baseline questionnaires. They were given contact details of the trial coordinator at baseline and at each follow-up assessment to ask any questions. Data were recorded in REDCap (Research Electronic Data Capture; hosted at the University of Queensland) [23,24]. In addition to primary and secondary outcomes, baseline data included duration of the current pain episode, demographics (age, gender, height, mass, education, job, and job status), and details about their low back symptoms (including location, intensity, duration, frequency, and past treatments; see Table 1 for specific questions and response options).

The participants were sent two reminders to complete the baseline data. Primary and secondary outcomes were collected at baseline and at 1, 3, 6, and 12 months after randomization. Pain intensity (pain visual analogue scale; see *Secondary Outcomes*), websites visited, and treatments used were recorded weekly until 3 months (primary endpoint) and then monthly until 12 months. Outcome data at each time point were collected using REDCap (automated email reminders), and up to three reminders were sent. To encourage retention, participants were entered into a draw for an iPad mini on completion of all data collection.

**Table 1:** Baseline characteristics of participants by group.

Characteristics			Group 1 (n=226)		Group 2 (n=214)
Acute^a^, n (%)			59 (26.1)		53 (24.8)
Chronic^b^, n (%)			167 (73.9)		161 (75.2)
Age at consent (years), mean (SD)			47.8 (14.1)		48.1 (14.0)
**Gender, n (%)**					
	Female	174 (77)		161 (75.2)	
	Male	49 (21.7)		53 (24.8)	
	Other	3 (1.3)		0 (0)	
Height (cm), mean (SD)			168.8 (10.1)		169.6 (9.3)
Mass (kg), mean (SD)			83.7 (20.1)		83.7 (20.7)
BMI (kg/m^2^), median (IQR)			28.9 (24.5-33.6)		27.7 (24.1-32.6)
**Level of education, n (%)**					
	High school certificate	38 (16.8)		30 (14)	
	Trade certificate	25 (11.1)		22 (10.3)	
	Diploma	37 (16.4)		29 (13.6)	
	Advanced diploma	12 (5.3)		13 (6.1)	
	Bachelor degree	59 (26.1)		57 (26.6)	
	Postgraduate degree	36 (15.9)		52 (24.3)	
	Other	19 (8.4)		11 (5.1)	
**Employment status, n (%)**					
	Full time/full duties	77 (34.1)		81 (37.9)	
	Full time/selected duties	8 (3.5)		2 (0.9)	
	Part time/full duties	33 (14.6)		43 (20.1)	
	Part time/selected duties	22 (9.7)		9 (4.2)	
	Not working/unemployed	4 (1.8)		6 (2.8)	
	Not working/employed/retraining	2 (0.9)		1 (0.5)	
	Not working/unemployed/retraining	4 (1.8)		7 (3.3)	
	Not working/unemployed	13 (5.8)		12 (5.6)	
	Not seeking employment	63 (27.9)		53 (24.8)	
Aboriginal/Torres Strait Islander, n (%)			3 (1.3)		3 (1.4)
Born in Australia, n (%)			159 (70.4)		161 (75.2)
First experience of LBP^c^, n (%)			14 (6.2)		12 (5.6)
**Number of episodes of LBP, n (%)**					
	1-5	24 (11.3)		16 (8)	
	5-10	16 (7.5)		23 (11.4)	
	10-15	11 (5.2)		10 (5)	
	15-20	7 (3.3)		14 (7)	
	More than 20	63 (29.7)		54 (26.9)	
	I am never without LBP	91 (42.9)		84 (41.8)	
Ever been given a diagnosis of LBP, n (%)			128 (56.6)		125 (58.4)
Have pain or altered sensation in buttocks/legs, n (%)			166 (73.5)		155 (72.4)
**Had problems with your bowel or bladder function since your back pain started, n (%)**					
	Bladder	21 (9.3)		17 (7.9)	
	Bowel	22 (9.7)		15 (7)	
	Bladder and bowel	30 (13.3)		22 (10.3)	
	No	153 (67.7)		160 (74.8)	
Had treatment for current episode of LBP, n (%)			142 (62.8)		116 (54.2)
Had treatments for previous episodes of LBP, n (%)			174 (82.5)		166 (82.6)
Have other medical conditions, n (%)			141 (62.4)		134 (62.6)
Have pain in other part of spine, n (%)			134 (59.3)		132 (61.7)
Feel pain in other areas of body, n (%)			143 (63.3)		130 (60.7)

^a^<6 weeks duration following period of no pain for at least 4 weeks.

^b^≥6 weeks duration.

^c^LBP: low back pain.

### Randomization and Allocation Concealment

The study biostatistician prepared the randomization schedule using stratification permuted block randomization, with block sizes of 6 to 12 stratified by symptom duration (acute LBP: <6 weeks duration with at least 4 weeks interval from a preceding episode; chronic LBP: all other presentations). After completion of baseline data, participants were randomized sequentially according to the schedule and provided with information regarding their mode of internet access.

### Blinding

Participants and investigators (except the project manager) were blinded to treatment allocation. All participants were advised in the participant information sheet that the study aimed to investigate the impact of internet use on their LBP. To maintain blinding, all participants recorded the address of any website visited for information regarding LBP over 12 months. The intervention group had access to the password-protected MyBackPain website. The control group was not aware of this website and was unable to access it until the website was launched to the public on July 30, 2019, after the primary endpoint had passed for all participants. Given that all outcome measures were self-reported, outcome assessments were also blinded. A blinded biostatistician conducted the data analysis.

### Study Treatments

#### Treatment

Participants randomized to the intervention group were given access to the MyBackPain website and encouraged to use it. The evidence-based content and framework of the MyBackPain website were developed according to consultation and collaboration with individuals with LBP, clinicians, and an international expert team [21].

Website development involved distillation of the highest quality information for acute and chronic LBP into easily understood resources in multiple formats. Textbox 1 lists the overarching principles.

Website access required a unique username and password provided to the intervention group participants. As we intended to study the natural use of the website, participants were free to determine how and when they accessed it and the content they used. Participants could use the website in multiple ways: self-directed browsing; automated, guided content tailored to the features of their presentation (evidence-based algorithms based on a pick-up tool for acute LBP [25] or STarT Back for chronic LBP [26]); and to *opt-in* for emails of key messages about LBP.

**Four overarching principles of the MyBackPain website.**

**Principles**

- To enhance consumer confidence to manage their condition and make evidence-based treatment choices and avoid ineffective, unnecessary or harmful investigations and treatments
- To demedicalize and normalize low back pain (LBP) with messages that reinforce that back pain is a natural part of life for many and in most cases can be managed with early return to activity
- To provide tools for individuals to identify the necessity for further investigation, management, or both
- To engage users in healthy behaviors and attitudes to reduce the burden of LBP

#### Control

Participants randomized to the control group were asked to use the internet to obtain information about LBP in a self-directed manner and record the address of any relevant websites weekly (weeks 1-12) and monthly (months 3-12) web-based diaries. They did not have access to or knowledge of the MyBackPain website, at least until after the primary endpoint. We cannot exclude the possibility that some control participants might have become aware of and used the site after its launch. At launch, 89 control participants were yet to complete a long-term follow-up.

### Outcome Measures

#### Primary Outcome

We used dimensions 2 and 3 of the Health Literacy Questionnaire (HLQ) [27] as coprimary outcomes to determine the extent to which participants considered “having sufficient information to manage my health” and “actively managing my health,” respectively, at 3 months after randomization. The validated HLQ includes 44 items in nine dimensions, with each dimension considered individually [27]. Dimensions 2 and 3 included four items assessed using a 4-point Likert scale (1=strongly disagree; 4=strongly agree), converted to a score of 0 to 100 for analysis. The survey’s preamble asked participants to consider their LBP when answering.

#### Secondary Outcomes

Secondary outcomes were (1) other HLQ dimensions, (2) quality of treatment choices, and (3) clinical outcomes. Secondary measures from the HLQ were dimensions: 1 (“feeling understood and supported by healthcare providers”) and 4-9 (“social support for health;” “appraisal of health information;” “ability to actively engage with healthcare providers;” “navigating the healthcare system;” “ability to find good health information” and “understand health information well enough to know what to do”), assessed using 4-point (dimensions 1 and 4-5) or 5-point (dimensions 6-9) Likert scales.

The stated and observed quality of treatment choices was assessed in three ways:

1. Quality of treatment preference (stated): participants indicated on a 5-item scale (effective, somewhat effective, unsure, not very effective, and not effective) the degree to which they considered a subset of 10 treatments discussed in the MyBackPain website to be effective for LBP in response to the question “Do you think these treatments are effective for people’s back pain? Note: think broadly about back pain, not just about your own.” Treatment choices were scored against the recommendations provided in the MyBackPain website according to the classifications of “good evidence,” “may work,” “not enough evidence,” “unlikely to work,” and “may be harmful” (Multimedia Appendix 1 shows the scoring matrix; scores from −20 to +22; a 1-point change would relate to a shift from being *unsure* of the efficacy of one treatment that is *unlikely to work* to considering it to be *not very effective*).
2. Quality of treatment preference (observed-scored): treatments used by participants (diary recording) were evaluated against the recommendations provided on the MyBackPain website. Each reported treatment was scored according to Multimedia Appendix 1 and summed (no upper and lower limits).
3. Quality of treatment preference (observed-proportion): the proportion of participants who chose treatments that are, according to MyBackPain, either recommended (“good evidence” and “may work”) or considered to have no effect or be harmful (“not enough evidence,” “unlikely to work,” and “may be harmful”) were assessed separately. Using diary data, participants were allocated a score of 1 if they used at least one recommended treatment and separately if they used a treatment that was harmful or had no effect. Proportions with scores of 1 were calculated.

LBP clinical outcomes were measured with the following validated tools: pain—visual analogue scale of average LBP in the last week (“no pain” at 0 and “worst pain imaginable” at 100); disability—Roland Morris Disability Questionnaire [28] (scores from 0 to 24; higher scores indicate more disability); and quality of life—Assessment of Quality of Life–8 dimension (utility scores from 0.0 to 1.0, with higher scores indicating better quality of life).

### Adherence to Intervention

We were unable to track the individual use of MyBackPain because of privacy concerns of the host sites (Arthritis Australia). We tracked the overall use of the site (number of sessions; new or returning users) using the Opentracker software.

### Sample Size

Sample size calculation was based on an effect size of 0.30 for the coprimary outcomes. A sample size of 440 participants (a minimum of 110/440, 25% with acute LBP) was required to achieve 80% power to detect the desired effect size, type 1 error of 0.05, and allowing for a 20% loss to follow-up at 3 months.

### Statistical Analysis

Data were analyzed using Stata version 16.0 (StataCorp) and included all participants in their randomized groups (intention-to-treat). The baseline characteristics of participants who did and did not provide primary outcomes were compared using two-tailed *t* tests or chi-square tests. Missing continuous outcomes were imputed using chained equations with predictive mean matching and five nearest neighbors, and missing binary outcomes were imputed using logistic regression imputation models with chained equations. The data were imputed separately for each randomized group. Imputation models for continuous outcomes included all baseline variables and all outcome variables, where possible. Imputation models for binary variables omitted binary baseline variables and outcome variables because of the potential for perfect prediction, including only continuous baseline variables. Estimates from 40 imputed data sets were combined using the Rubin rules [29].

For the primary outcomes and continuous secondary outcomes, mean differences between groups were estimated at each time point using longitudinal linear mixed-effects models. These models included all data from 1, 3 (primary endpoint), 6, and 12 months as outcomes for each participant. The models included an interaction between month and randomized group as a fixed effect and random intercepts for participants. Models were adjusted for baseline values of the outcome and stratification variables (symptom duration). Similar longitudinal logistic regression models were used for secondary binary outcomes, with results presented as odds ratios and risk ratios at each time point. Model assumptions and the validity of the imputed data sets were assessed using standard diagnostic plots. No statistical adjustments were made for multiple testing. Complete case analyses were also performed.

## Results

**Figure 1:**
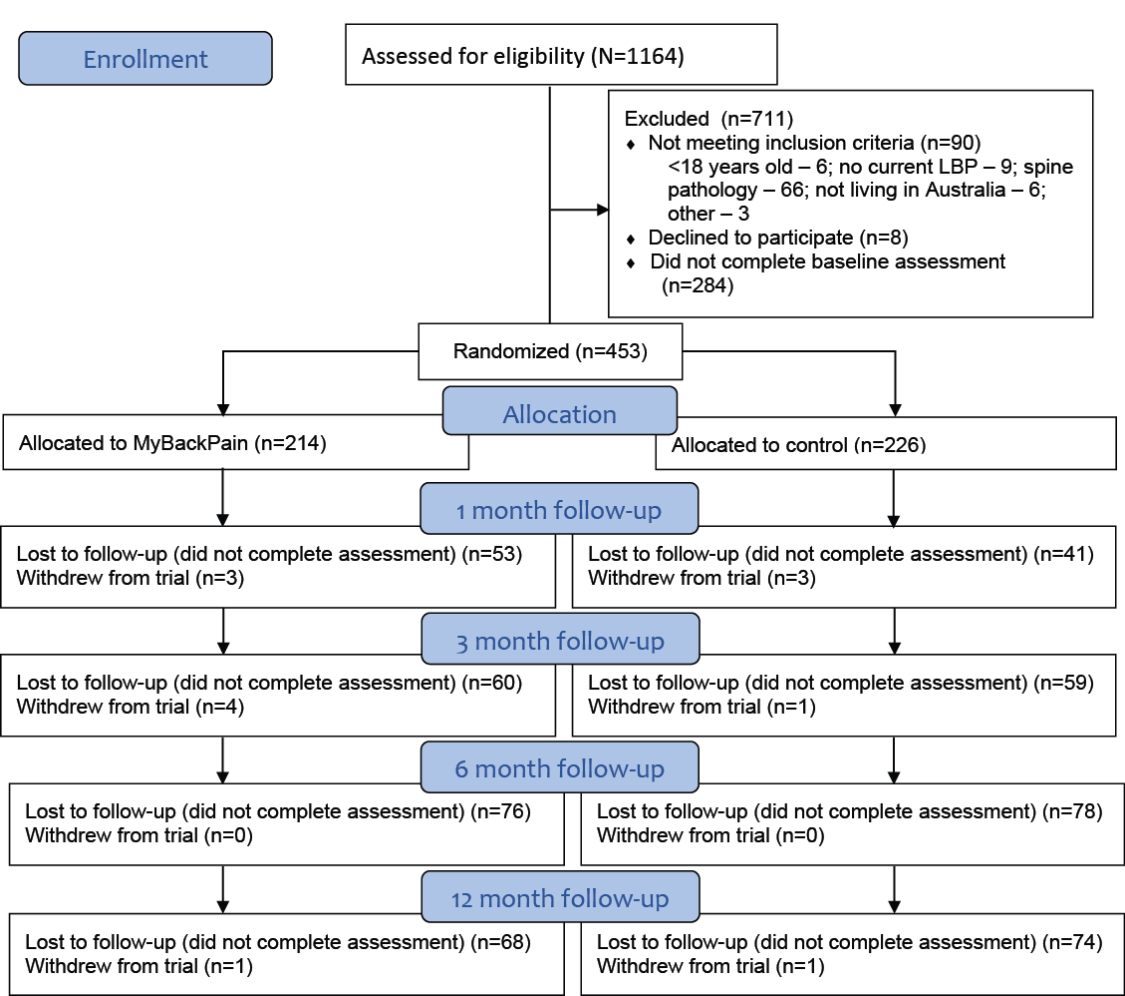
CONSORT (Consolidated Standards of Reporting Trails) flow diagram. LBP: low back pain.

Recruitment was conducted from December 6, 2017, to January 16, 2019, with follow-up completed on January 16, 2020. We enrolled 453 participants who completed the baseline assessment (Figure 1). In total, 13 withdrew (different time points), and their data were excluded from the analysis. The groups were similar at baseline (Multimedia Appendix 2). Loss to follow-up at the primary endpoint (3 months) was 26.1% (59/226) in the control group and 28% (60/214) in the MyBackPain group. Those lost to follow-up were younger and had fewer previous episodes of LBP (Multimedia Appendix 2).

For the primary outcomes at 3 months, between-group differences in dimensions 2 (mean difference −0.87 units, 95% CI −3.56 to 1.82) and 3 (mean difference −0.41 units, 95% CI −2.78 to 1.96) of the HLQ were small with wide CIs (Multimedia Appendix 3).

For secondary outcomes, the MyBackPain group scored greater for treatment choice (stated) than the control group at 1 month (−0.91 units, 95% CI 0.16 to 1.67), but differences beyond that time point were of inconsistent direction and unlikely to be clinically significant (Multimedia Appendix 3), as was the observed proportion of participants selecting a recommended treatment at all time points (Table 2). Between-group differences in other secondary outcomes (other HLQ dimensions and clinical outcomes) at any time point were of inconsistent direction.

The results for the complete case analysis were similar (Multimedia Appendices 4 and 5), except that more participants (observed-proportion) in the treatment group were likely to select a recommended treatment (odds ratio 5.76, 95% CI 1.01 to 32.71; risk ratio 1.29, 95% CI 1.01 to 1.58) at 3 months (Table 2).

Up to the launch date (after the primary endpoint for all participants), the MyBackPain website was accessed an average of 3.4 times, each by 183 unique users. This represented 85.5% (183/214) of the patients allocated to the MyBackPain group.

**Table 2:** Participants choosing treatment types (the binary outcomes)^a^.

Treatment type and month			Control							MyBackPain							Risk ratio (95% CI)			*P* value
			Total, participant, n		Participant, n (%)		Number missing (n=226), n (%)		Total, participant, n			Participant, n (%)		Number missing (n=214), n (%)						
**Observed treatment choice harmful or no effect**																				
	1	83		20 (24.1)		143 (63.2)		63			17 (26.9)		151 (70.6)		1.07 (0.55-1.59)			.79		
	3	73		12 (16.4)		153 (67.7)		52			13 (25)		162 (75.7)		1.39 (0.56-2.23)			.35		
	6	67		11 (16.4)		159 (70.3)		67			11 (16.4)		147 (68.7)		0.76 (0.30-1.22)			.30		
	12	89		18 (20.2)		137 (60.6)		72			10 (13.8)		142 (66.3)		0.94 (0.40-1.48)			.83		
**Observed treatment choice recommended**																				
	1	83		55 (66.2)		143 (63.2)		63			40 (63.5)		151 (70.6)		0.89 (0.69-1.08)			.26		
	3	73		49 (67.1)		153 (67.7)		52			37 (71.1)		162 (75.7)		0.99 (0.78-1.20)			.94		
	6	67		54 (80.6)		159 (70.3)		67			47 (70.1)		147 (68.7)		0.84 (0.64-1.05)			.13		
	12	89		65 (73)		137 (60.6)		72			54 (75)		142 (66.3)		0.99 (0.78-1.20)			.91		

^a^Odds and risk ratios are calculated using multiply imputed data. Counts and percentages calculated using the observed data only.

## Discussion

### Principal Findings

The results of this study showed that the natural use of a web-based information-only resource (MyBackPain [20]) did not improve the two dimensions of health literacy more than self-guided access to web-based LBP information. Use of the website did not achieve better outcomes for other secondary measures of clinical features of pain, disability, quality of life, or other aspects of health literacy, except for the stated and observed selection of recommended treatments at one follow-up time point.

### Interpretation of Findings

MyBackPain is a website for individuals with LBP that was developed with extensive consumer input to ensure that content and design are aligned with patient needs and preferences [21]. The website’s intention is to enhance an individual’s capacity or confidence to manage their condition, including decision support for the selection of effective treatments. The primary outcomes for this randomized controlled trial were selected to reflect this goal with respect to “having sufficient information to manage my health” or “actively managing my health.” Contrary to our hypothesis, the use of the MyBackPain website did not achieve greater improvement in these domains than the unguided use of the internet. However, the finding that access to MyBackPain achieved greater improvement in stated treatment choices at 1 month provides preliminary evidence for the improvement of active management of their condition.

Health literacy is defined as “the cognitive and social skills which determine the motivation and ability of individuals to gain access to, understand and use information in ways which promote and maintain good health” [30]. Health literacy extends beyond the provision of information to the empowerment of individuals to use information [27]. The use of MyBackPain did not more effectively modify an individual’s *perception* of their ability to manage their own health than unguided use of the internet, but the data suggest some short-term improvement in treatment choices. One interpretation is that improvement in these elements of health literacy may be satisfied by the general use of information on the internet but that the quality of the resulting actions (ie, treatment selection) depends on the quality of the information provided. The control group’s lower treatment choice scores concur with a recent review that identified only 43.3% of treatment recommendations provided by freely accessible websites were accurate [17]. The treatment recommendations provided in MyBackPain are based on expert panel consensus.

On the basis of population data for the HLQ domains from the Australian Bureau of Statistics, our sample had similar health literacy at baseline (dimension 2: our data=mean 3.12, SD 0.6, vs Australian Bureau of Statistics data for individuals who report a *back problem*=mean 3.13, relative SE 0.5; dimension 3: 3.23, SD 0.6, vs 3.03, SE 0.6). However, our sample had low quality of life utility scores compared with Australian norms (our data: mean 0.55, SD 0.22, vs mean 0.799, 95% CI 0.792-0.806 [31]). Despite the co-design of MyBackPain in individuals with LBP, the presentation of information might not be optimal for those with low quality of life [32].

MyBackPain was designed as an information resource. The information is provided in multiple formats but with limited interactivity. Benefits might be enhanced by greater interactivity, such as inclusion of a web-based discussion group [33], regular action plans [34], or provision of active intervention. Refinement of MyBackPain would require assessment because recent evidence highlights inconsistent evidence for the efficacy of web-based cognitive behavioral therapies for LBP [18,19,35,36], interactivity [12], and personalization [36,37].

### Treatment Selection

This study provides preliminary evidence that access to MyBackPain might achieve short-term improvement in treatment choices. The largest component of the website, as requested by consumers [10], is a treatment comparison tool. This tool provides summaries of the treatment, the quality of evidence for efficacy, and pros and cons for its use with an evidence icon that provides a quick reference of the level of evidence and effectiveness [21]. Summaries were developed with input from an international expert team and consumers [21]. Although no other studies have tested the effectiveness of web-based information to improve treatment choices, studies that are not specific to LBP have reported reduced health care usage (improved self-management and decreased use of ineffective treatments) related to high-quality, evidence-based, web-based information [38].

We did not include treatment selection as a primary outcome because of the novelty of our measures. The measures were tested for comprehension in individuals with LBP but had unknown psychometric properties. The *observed* and *stated* measures of treatment choice were based on expert consensus recommendations in the website. Alignment of stated and observed choices with the recommended treatments resulted in a higher score. Better stated treatment choices by the MyBackPain group at 1 month provide stimulus for further work. No group difference at 3 months implies that sustained improvement might require sustained engagement with the website.

### Web Resource Evaluation

It is recommended that information resources be tested for the credibility and accuracy of content [39]. According to these criteria, the MyBackPain website has a strong foundation supported by research with consumers [32] and expert consultation for content accuracy [21,40]. Multimedia Appendix 6 [10,21,25,32,41-43] considers MyBackPain against evidence standards for digital health technologies [41].

### Limitations

There are several limitations that need to be considered. First, there was a large loss to follow-up, which is common in web-based trials [35,44]. Although multiple imputations were implemented, if participants were lost to follow-up due to worsening of their condition, this analysis may not be appropriate. Second, the individual use of MyBackPain could not be tracked. That 14.5% (31/214) of the treatment group did not access MyBackPain is disappointing, but it is important to note that the trial was pragmatic and designed to evaluate natural use.

### Conclusions

This is the first randomized controlled trial of an information resource for LBP to test its impact on health literacy, quality of treatment choices, and clinical outcomes. Unlike most websites for LBP, MyBackPain was specifically designed to meet the needs of individuals with LBP and included a focus on treatment choices. The results provided no evidence of differences between groups for the primary outcomes related to health literacy. There is limited preliminary evidence of the impact of empowerment on making informed choices for the management of their condition, which is the intention of improved health literacy. Loss to follow-up should be considered when interpreting these results. Future work should consider the potential to enhance impact through the addition of interactivity and interventions and greater support provided to users to engage with the resource.

## Appendix

Multimedia Appendix 1 Scoring matrix for evaluation of quality of treatment preference.

Multimedia Appendix 2 Baseline characteristics and outcome scores of participants who did and did not report both primary outcomes, reported as number (percentage) unless otherwise stated.

Multimedia Appendix 3 Mean (SD) of continuous outcomes and mean difference (95% CI) between MyBackPain and control groups over the 12-month study period.

Multimedia Appendix 4 Complete case data: mean (SD) of continuous outcomes and mean difference between groups over time (MyBackPain and control).

Multimedia Appendix 5 Complete case data: number (percentage) of participants choosing treatment types (binary outcomes).

Multimedia Appendix 6 Evidence for effectiveness standards.

Multimedia Appendix 7 CONSORT-eHEALTH checklist (V 1.6.1).

## Abbreviations

HLQ: Health Literacy Questionnaire
LBP: low back pain
REDCap: Research Electronic Data Capture

## References

[1] GBD 2017 Disease Injury Incidence Prevalence Collaborators (2018). Global, regional, and national incidence, prevalence, and years lived with disability for 354 diseases and injuries for 195 countries and territories, 1990-2017: a systematic analysis for the Global Burden of Disease Study 2017. Lancet.

[2] Buchbinder R, van Tulder M, Öberg B, Costa LM, Woolf A, Schoene M, Croft P, Lancet Low Back Pain Series Working Group (2018). Low back pain: a call for action. Lancet.

[3] Alyousef B, Cicuttini FM, Davis SR, Bell R, Botlero R, Urquhart DM (2018). Negative beliefs about back pain are associated with persistent, high levels of low back disability in community-based women. Menopause.

[4] Carey M, Turon H, Goergen S, Sanson-Fisher R, Yoong SL, Jones K (2015). Patients' experiences of the management of lower back pain in general practice: use of diagnostic imaging, medication and provision of self-management advice. Aust J Prim Health.

[5] Clarke MA, Moore JL, Steege LM, Koopman RJ, Belden JL, Canfield SM, Meadows SE, Elliott SG, Kim MS (2016). Health information needs, sources, and barriers of primary care patients to achieve patient-centered care: a literature review. Health Informatics J.

[6] Kummervold PE, Chronaki CE, Lausen B, Prokosch H, Rasmussen J, Santana S, Staniszewski A, Wangberg SC (2008). eHealth trends in Europe 2005-2007: a population-based survey. J Med Internet Res.

[7] Fox S (2009). The social life of health information. Washington DC.

[8] Volkman JE, Luger TM, Harvey KL, Hogan TP, Shimada SL, Amante D, McInnes DK, Feng H, Houston TK (2014). The National Cancer Institute's Health Information National Trends Survey [HINTS]: a national cross-sectional analysis of talking to your doctor and other healthcare providers for health information. BMC Fam Pract.

[9] Lee K, Hoti K, Hughes JD, Emmerton L (2014). Dr Google and the consumer: a qualitative study exploring the navigational needs and online health information-seeking behaviors of consumers with chronic health conditions. J Med Internet Res.

[10] Nielsen M, Jull G, Hodges PW (2014). Information needs of people with low back pain for an online resource: a qualitative study of consumer views. Disabil Rehabil.

[11] Li N, Orrange S, Kravitz RL, Bell RA (2014). Reasons for and predictors of patients' online health information seeking following a medical appointment. Fam Pract.

[12] Hewitt S, Sephton R, Yeowell G (2020). The effectiveness of digital health interventions in the management of musculoskeletal conditions: systematic literature review. J Med Internet Res.

[13] Butler L, Foster NE (2003). Back pain online: a cross-sectional survey of the quality of web-based information on low back pain. Spine (Phila Pa 1976).

[14] Hendrick PA, Ahmed OH, Bankier SS, Chan TJ, Crawford SA, Ryder CR, Welsh LJ, Schneiders AG (2012). Acute low back pain information online: an evaluation of quality, content accuracy and readability of related websites. Man Ther.

[15] Li L, Irvin E, Guzmán J, Bombardier C (2001). Surfing for back pain patients: the nature and quality of back pain information on the Internet. Spine (Phila Pa 1976).

[16] Costa N, Nielsen M, Jull G, Claus AP, Hodges PW (2019). Low back pain websites do not meet the needs of consumers: a study of online resources at three time points. Health Inf Manag.

[17] Ferreira G, Traeger AC, Machado G, O'Keeffe M, Maher CG (2019). Credibility, accuracy, and comprehensiveness of internet-based information about low back pain: a systematic review. J Med Internet Res.

[18] Buhrman M, Nilsson-Ihrfeldt E, Jannert M, Ström L, Andersson G (2011). Guided internet-based cognitive behavioural treatment for chronic back pain reduces pain catastrophizing: a randomized controlled trial. J Rehabil Med.

[19] Carpenter KM, Stoner SA, Mundt JM, Stoelb B (2012). An online self-help CBT intervention for chronic lower back pain. Clin J Pain.

[20] MyBackPain.

[21] Hodges PW, Setchell J, Nielsen M (2020). An internet-based consumer resource for people with low back pain (MyBackPain): development and evaluation. JMIR Rehabil Assist Technol.

[22] Hall LM, Ferreira M, Setchell J, French S, Kasza J, Bennell KL, Hunter D, Vicenzino B, Dickson C, Hodges P (2019). MyBackPain-evaluation of an innovative consumer-focused website for low back pain: study protocol for a randomised controlled trial. BMJ Open.

[23] Harris PA, Taylor R, Minor BL, Elliott V, Fernandez M, O'Neal L, McLeod L, Delacqua G, Delacqua F, Kirby J, Duda SN, REDCap Consortium (2019). The REDCap consortium: building an international community of software platform partners. J Biomed Inform.

[24] Harris PA, Taylor R, Thielke R, Payne J, Gonzalez N, Conde JG (2009). Research electronic data capture (REDCap) - a metadata-driven methodology and workflow process for providing translational research informatics support. J Biomed Inform.

[25] Traeger AC, Henschke N, Hübscher M, Williams CM, Kamper SJ, Maher CG, Moseley GL, McAuley JH (2016). Estimating the risk of chronic pain: development and validation of a prognostic model (PICKUP) for patients with acute low back pain. PLoS Med.

[26] Hill JC, Dunn KM, Lewis M, Mullis R, Main CJ, Foster NE, Hay EM (2008). A primary care back pain screening tool: identifying patient subgroups for initial treatment. Arthritis Rheum.

[27] Osborne RH, Batterham RW, Elsworth GR, Hawkins M, Buchbinder R (2013). The grounded psychometric development and initial validation of the Health Literacy Questionnaire (HLQ). BMC Public Health.

[28] Roland M, Morris R (1983). A study of the natural history of low-back pain. Part II: development of guidelines for trials of treatment in primary care. Spine (Phila Pa 1976).

[29] Carpenter J, Kenward M (2013). Multiple Imputation and its Application, 1st Edition.

[30] (1998). Health promotion glossary. World Health Organization.

[31] Maxwell A, Özmen M, Iezzi A, Richardson J (2016). Deriving population norms for the AQoL-6D and AQoL-8D multi-attribute utility instruments from web-based data. Qual Life Res.

[32] Setchell J, Turpin M, Costa N, Hodges P (2020). Web-based consumer health education about back pain: findings of potential tensions from a photo-elicitation and observational study. JMIR Rehabil Assist Technol.

[33] Maclachlan LR, Mills K, Lawford BJ, Egerton T, Setchell J, Hall LM, Plinsinga ML, Besomi M, Teo PL, Eyles JP, Mellor R, Melo L, Robbins S, Hodges PW, Hunter DJ, Vicenzino B, Bennell KL (2020). Design, delivery, maintenance, and outcomes of peer-to-peer online support groups for people with chronic musculoskeletal disorders: systematic review. J Med Internet Res.

[34] Riva S, Camerini A, Allam A, Schulz PJ (2014). Interactive sections of an internet-based intervention increase empowerment of chronic back pain patients: randomized controlled trial. J Med Internet Res.

[35] Garg S, Garg D, Turin TC, Chowdhury MF (2016). Web-based interventions for chronic back pain: a systematic review. J Med Internet Res.

[36] Irvine AB, Russell H, Manocchia M, Mino DE, Cox GT, Morgan R, Gau JM, Birney AJ, Ary DV (2015). Mobile-Web app to self-manage low back pain: randomized controlled trial. J Med Internet Res.

[37] Lustria ML, Cortese J, Noar SM, Glueckauf RL (2009). Computer-tailored health interventions delivered over the web: review and analysis of key components. Patient Educ Couns.

[38] Spoelman WA, Bonten TN, de Waal MW, Drenthen T, Smeele IJ, Nielen MM, Chavannes NH (2016). Effect of an evidence-based website on healthcare usage: an interrupted time-series study. BMJ Open.

[39] Greaves F, Joshi I, Campbell M, Roberts S, Patel N, Powell J (2019). What is an appropriate level of evidence for a digital health intervention?. Lancet.

[40] French SD, Nielsen M, Hall LM, Nicolson PJ, van Tulder M, Bennell KL, Hinman RS, Maher CG, Jull G, Hodges PW (2019). Essential key messages about diagnosis, imaging, and self-care for people with low back pain: a modified Delphi study of consumer and expert opinions. Pain.

[41] (2018). Evidence standards framework for digital health technologies. National Institute for Health and Care Excellence.

[42] Nielsen M, Jull G, Hodges PW (2016). Designing an online resource for people with low back pain: health-care provider perspectives. Aust J Prim Health.

[43] Hill JC, Whitehurst DG, Lewis M, Bryan S, Dunn KM, Foster NE, Konstantinou K, Main CJ, Mason E, Somerville S, Sowden G, Vohora K, Hay EM (2011). Comparison of stratified primary care management for low back pain with current best practice (STarT Back): a randomised controlled trial. Lancet.

[44] Weymann N, Dirmaier J, von Wolff A, Kriston L, Härter M (2015). Effectiveness of a web-based tailored interactive health communication application for patients with type 2 diabetes or chronic low back pain: randomized controlled trial. J Med Internet Res.

