# Supplementary material for: Effect of a Consumer-Focused Website for Low Back Pain on Health Literacy, Treatment Choices, and Clinical Outcomes: Randomized Controlled Trial
Source: J Med Internet Res. 2021 Jun 15;23(6):e27860. doi: 10.2196/27860 (PMC8277358; doi:10.2196/27860)
Supplement: Multimedia Appendix 1 [file jmir_v23i6e27860_app1.docx]

**Multimedia Appendix 1** Scoring matrix for evaluation of quality of treatment preference

|  | **Patient decision from 5-point scale** | | | | |
| --- | --- | --- | --- | --- | --- |
|  | Effective | Somewhat effective | Unsure | Not very effective | Not effective |
| **Recommendation from MyBackPain website** |  |  |  |  |  |
| Good evidence | 4 | 2 | 0 | -2 | -4 |
| May work | 2 | 1 | 0 | -1 | -2 |
| Not enough evidence | 0 | 0 | 1 | 0 | 0 |
| Unlikely to work | -2 | -1 | 0 | 1 | 2 |
| May be harmful | -4 | -2 | 0 | 2 | 4 |

Interpretation note: A 1-point change in this score would relate to a shift from being “unsure” of the efficacy of one treatment that is *unlikely to work* to considering it to be “not very effective”; a 2-point change would equate to perception of efficacy of a treatment with good evidence to change from “somewhat effective” to “effective".
