# Supplementary material for: Effect of a Consumer-Focused Website for Low Back Pain on Health Literacy, Treatment Choices, and Clinical Outcomes: Randomized Controlled Trial
Source: J Med Internet Res. 2021 Jun 15;23(6):e27860. doi: 10.2196/27860 (PMC8277358; doi:10.2196/27860)
Supplement: Multimedia Appendix 2 [file jmir_v23i6e27860_app2.docx]

**Multimedia Appendix 2** Baseline characteristics and outcome scores of participants who did and did not report both primary outcomes, reported as number (percentage) unless otherwise stated.

| **Characteristic** | **Did not provide both primary outcomes (n=119)** | **Provided both primary outcomes (n=321)** | **p-value^a^** |
| --- | --- | --- | --- |
| Group |  |  | 0.65 |
| Control | 59 (49.6%) | 167 (52.0%) |  |
| MyBackPain | 60 (50.4%) | 154 (48.0%) |  |
| Acute/Chronic |  |  | 0.36 |
| Acute | 34 (28.6%) | 78 (24.3%) |  |
| Chronic | 85 (71.4%) | 243 (75.7%) |  |
| Age at consent (years), mean (SD) | 44.5 (14.1) | 49.2 (13.8) | 0.002 |
| Gender |  |  | 0.52 |
| Female | 93 (78.2%) | 242 (75.4%) |  |
| Male | 26 (21.8%) | 76 (23.7%) |  |
| Other | 0 (0.0%) | 3 (0.9%) |  |
| Height (cm), mean (SD) | 169.6 (10.1) | 169.1 (9.6) | 0.62 |
| Mass (kg), mean (SD) | 82.9 (19.1) | 84.0 (20.8) | 0.63 |
| BMI (kg/m2), median (IQR) | 27.4 (23.4-33.9) | 28.6 (24.6-32.8) | 0.59 |
| Level of education |  |  | 0.83 |
| High School Certificate | 19 (16.0%) | 49 (15.3%) |  |
| Trade Certificate | 13 (10.9%) | 34 (10.6%) |  |
| Diploma | 14 (11.8%) | 52 (16.2%) |  |
| Advanced Diploma | 9 (7.6%) | 16 (5.0%) |  |
| Bachelor Degree | 31 (26.1%) | 85 (26.5%) |  |
| Postgraduate Degree | 23 (19.3%) | 65 (20.2%) |  |
| Other | 10 (8.4%) | 20 (6.2%) |  |
| Employment status |  |  | 0.45 |
| Full time/full duties | 50 (42.0%) | 108 (33.6%) |  |
| Full time/selected duties | 4 (3.4%) | 6 (1.9%) |  |
| Part time/full duties | 23 (19.3%) | 53 (16.5%) |  |
| Part time/selected duties | 8 (6.7%) | 23 (7.2%) |  |
| Not working/unemployed | 1 (0.8%) | 9 (2.8%) |  |
| Not working/employed/retraining | 1 (0.8%) | 2 (0.6%) |  |
| Not working/unemployed/retraining | 3 (2.5%) | 8 (2.5%) |  |
| Not working/unemployed | 4 (3.4%) | 21 (6.5%) |  |
| Not seeking employment | 25 (21.0%) | 91 (28.3%) |  |
| Aboriginal/Torres Strait Islander | 4 (3.4%) | 2 (0.6%) | 0.028 |
| Born in Australia | 90 (75.6%) | 230 (71.7%) | 0.41 |
| First experience of low back pain | 9 (7.6%) | 17 (5.3%) | 0.37 |
| Number of episodes of low back pain |  |  | 0.046 |
| 1-5 episodes | 13 (11.8%) | 27 (8.9%) |  |
| 5-10 episodes | 18 (16.4%) | 21 (6.9%) |  |
| 10-15 episodes | 4 (3.6%) | 17 (5.6%) |  |
| 15-20 episodes | 7 (6.4%) | 14 (4.6%) |  |
| More than 20 episodes | 29 (26.4%) | 88 (29.0%) |  |
| I am never without low back pain | 39 (35.5%) | 136 (44.9%) |  |
| Ever been given a diagnosis of low back pain | 64 (53.8%) | 189 (58.9%) | 0.34 |
| Have pain or altered sensation in buttocks/legs | 88 (73.9%) | 233 (72.6%) | 0.77 |
| Had problems with your bowel or bladder function since your back pain started |  |  | 0.78 |
| Bladder | 10 (8.4%) | 28 (8.7%) |  |
| Bowel | 10 (8.4%) | 27 (8.4%) |  |
| Bladder and bowel | 11 (9.2%) | 41 (12.8%) |  |
| No | 88 (73.9%) | 225 (70.1%) |  |
| Had treatment for current episode of low back pain | 73 (61.3%) | 185 (57.6%) | 0.48 |
| Had treatments for previous episodes of low back pain | 88 (80.0%) | 252 (83.4%) | 0.42 |
| Have other medical conditions | 72 (60.5%) | 203 (63.2%) | 0.60 |
| Have pain in other part of spine | 71 (59.7%) | 195 (60.7%) | 0.84 |
| Feel pain in other areas of body | 73 (61.3%) | 200 (62.3%) | 0.85 |
| HLQ Scale 1 score (100-point scale), mean (SD) | 77.8 (15.3) | 78.2 (15.4) | 0.80 |
| HLQ Scale 2 score (100-point scale), mean (SD) | 70.6 (14.2) | 71.5 (13.9) | 0.57 |
| HLQ Scale 3 score (100-point scale), mean (SD) | 73.4 (15.4) | 74.8 (13.5) | 0.33 |
| HLQ Scale 4 score (100-point scale), mean (SD) | 67.1 (16.9) | 68.6 (14.3) | 0.33 |
| HLQ Scale 5 score (100-point scale), mean (SD) | 75.8 (12.3) | 77.0 (11.8) | 0.37 |
| HLQ Scale 6 score (100-point scale), mean (SD) | 75.1 (15.3) | 75.4 (14.8) | 0.84 |
| HLQ Scale 7 score (100-point scale), mean (SD) | 70.8 (15.3) | 71.8 (14.5) | 0.55 |
| HLQ Scale 8 score (100-point scale), mean (SD) | 80.1 (11.6) | 80.5 (11.6) | 0.74 |
| HLQ Scale 9 score (100-point scale), mean (SD) | 83.7 (11.3) | 85.0 (10.9) | 0.27 |
| Quality of Life Questionnaire (utility score), mean (SD) | 0.5 (0.2) | 0.6 (0.2) | 0.38 |
| Roland Morris Disability Questionnaire score, mean (SD) | 8.7 (5.9) | 9.0 (5.8) | 0.56 |
| Treatment Choices (Observed), mean (SD) | 2.7 (3.1) | 2.7 (2.9) | 0.97 |
| Treatment Choices (Stated), mean (SD) | 5.3 (4.7) | 5.2 (4.3) | 0.94 |
| Pain VAS, mean (SD) | 53.1 (19.0) | 53.0 (18.4) | 0.95 |
| Observed treatment choice harmful / no effect | 17 (23.6%) | 47 (25.4%) | 0.77 |
| Observed treatment choice recommended | 55 (76.4%) | 147 (79.5%) | 0.59 |
| ^a^P-values based on t-tests for continuous characteristics and chi-squared tests for categorical characteristics; BMI – body mass index. HLQ – Health Literacy Questionnaire (converted to 100 point scale); For conversion of HLQ data (0-100 scale) to conventional 1-4 scale = value*3/100+1. | | | |
