# Supplementary material for: Effect of a Consumer-Focused Website for Low Back Pain on Health Literacy, Treatment Choices, and Clinical Outcomes: Randomized Controlled Trial
Source: J Med Internet Res. 2021 Jun 15;23(6):e27860. doi: 10.2196/27860 (PMC8277358; doi:10.2196/27860)
Supplement: Multimedia Appendix 3 [file jmir_v23i6e27860_app3.docx]

| **Outcome** | **Baseline** | | **1 month** | | | | **3 months** | | | | **6 months** | | | | **12 months** | | | |
| --- | --- | --- | --- | --- | --- | --- | --- | --- | --- | --- | --- | --- | --- | --- | --- | --- | --- | --- |
|  | **Control^a^ (n=226)** | **MBC^b toc^ (n=214)** | **Mean difference (95%CI)** | | | ***P* value** | **Mean difference (95%CI)** | | ***P* value** | **Mean difference (95%CI)** | | | | ***P* value** | **Mean difference (95%CI)** | | | ***P* value** |
|  |  |  | **Control^d^** | | **MBP^e^** |  | **Control^f^** | **MBP^g^** |  | **Control^h^** | | | **MBP^i^** |  | **Control^i^** | | **MBP^k^** |  |
| **Primary outcomes** | | | | | | | | | | | | | | | | | | |
| HLQ D2^l^ (0-100)^m^ |  | | 0.56 (−2.03 to 3.15) | | | .67 | −0.87 (−3.56 to 1.82) | | .53 | | 2.11 (−0.75 to 4.97) | | | .15 | 0.41 (−2.46 to 3.29) | | | .78 |
|  | 70.8 (14.0) | 71.7 (14.0) | 71.8 (13.3) | 72.8 (13.9) | |  | 73.3 (12.8) | 72.9 (13.9) |  | | 73.4 (13.8) | 76.0 (14.0) | |  | 74.5 (14.3) | 75.3 (14.9) | |  |
| HLQ D3 (0-100)^m^ |  | | 0.81 (−1.55 to 3.16) | | | .50 | −0.41 (−2.78 to 1.96) | | .73 | | 2.02 (−0.51 to 4.56) | | | .12 | 1.52 (−1.19 to 4.23) | | | .27 |
|  | 74.3 (13.7) | 74.6 (14.5) | 73.7 (12.9) | 74.7 (13.1) | |  | 75.1 (13.1) | 74.9 (13.1) |  | | 74.4 (14.2) | 76.6 (13.4) | |  | 76.6 (12.5) | 78.3 (13.2) | |  |
| **Secondary outcomes** | | | | | | | | | | | | | | | | | | |
| Treatment Choices (Stated) (−20 to +22) |  | | 0.93 (0.03 to 1.84) | | | .04 | 0.36 (−0.59 to 1.31) | | .46 | | 0.87 (−0.13 to 1.86) | | | .09 | 0.59 (−0.36 to 1.53) | | | .22 |
|  | 5.3 (4.2) | 5.2 (4.6) | 5.1 (4.2) | 6.0 (4.9) | |  | 5.3 (4.9) | 5.7 (4.9) |  | | 5.2 (4.4) | 6.1 (5.1) | |  | 5.1 (4.5) | 5.7 (4.9) | |  |
| Treatment Choices (Observed) |  | | −0.32 (−1.07 to 0.42) | | | .39 | −0.13 (−1.18 to 0.92) | | .81 | | −1.10 (−1.98 to −0.22) | | | .01 | 0.02 (−0.65 to 0.69) | | | .95 |
|  | 3.1 (3.1) | 2.2 (2.7) | 2.0 (2.8) | 1.6 (2.3) | |  | 2.1 (3.0) | 1.9 (3.4) |  | | 3.3 (2.9) | 2.1 (2.7) | |  | 2.2 (2.7) | 2.2 (1.9) | |  |
| RMDQ^n^ (0-24) |  | | 0.35 (−0.77 to 1.46) | | | .54 | 0.46 (−0.64 to 1.55) | | .41 | | −0.15 (−1.37 to 1.07) | | | .81 | 0.06 (−1.14 to 1.27) | | | .92 |
|  | 9.3 (5.7) | 8.5 (6.0) | 7.1 (6.0) | 7.1 (5.9) | |  | 7.2 (6.2) | 7.2 (6.1) |  | | 7.3 (6.5) | 6.7 (6.4) | |  | 7.1 (6.2) | 6.7 (6.5) | |  |
| Quality of Life (utility score) (0-1) |  | | 0.01 (−0.03 to 0.04) | | | .72 | 0.00 (−0.03 to 0.04) | | .86 | | 0.01 (−0.03 to 0.04) | | | .75 | −0.00 (−0.04 to 0.03) | | | .89 |
|  | 0.54 (0.20) | 0.55 (0.23) | 0.54 (0.21) | 0.56 (0.22) | |  | 0.56 (0.21) | 0.58 (0.23) |  | | 0.56 (0.21) | 0.57 (0.23) | |  | 0.57 (0.23) | 0.58 (0.23) | |  |
| Pain VAS^o^ (0-100) |  | | −0.95 (−6.04 to 4.15) | | | .71 | 0.48 (−4.94 to 5.89) | | .86 | | 0.85 (−4.83 to 6.54) | | | .77 | −1.58 (−7.38 to 4.22) | | | .59 |
|  | 54.2 (18.2) | 51.8 (18.8) | 48.2 (23.8) | 46.3 (25.8) | |  | 46.8 (25.4) | 46.4 (25.9) |  | | 46.4 (25.6) | 46.3 (27.8) | |  | 48.2 (26.5) | 45.7 (27.2) | |  |
| HLQ D1 (0-100)^m^ |  | | 0.50 (−2.41 to 3.41) | | | .74 | 0.16 (−2.63 to 2.96) | | .91 | | 1.00 (−2.02 to 4.02) | | | .52 | 0.96 (−2.16 to 4.09) | | | .54 |
|  | 78.1 (15.2) | 78.0 (15.6) | 77.6 (14.9) | 78.1 (16.2) | |  | 77.6 (15.6) | 77.7 (14.6) |  | | 77.9 (15.4) | 78.8 (14.7) | |  | 78.4 (15.3) | 79.3 (15.0) | |  |
| HLQ D4 (0-100)^m^ |  | | −0.14 (−2.82 to 2.54) | | | .92 | −1.52 (−4.33 to 1.29) | | .29 | | −0.40 (−3.39 to 2.60) | | | .80 | −1.01 (−3.76 to 1.75) | | | .47 |
|  | 69.4 (14.9) | 67.0 (15.2) | 69.3 (15.1) | 67.7 (16.2) | |  | 70.8 (14.5) | 67.8 (14.1) |  | | 71.7 (16.5) | 69.8 (15.7) | |  | 72.5 (15.7) | 70.0 (16.4) | |  |
| HLQ D5 (0-100)^m^ |  | | 0.74 (−1.62 to 3.09) | | | .54 | −1.31 (−3.74 to 1.13) | | .29 | | −0.07 (−2.54 to 2.39) | | | .95 | −0.32 (−2.72 to 2.08) | | | .79 |
|  | 76.3 (12.5) | 77.1 (11.4) | 75.7 (11.2) | 76.9 (12.9) | |  | 77.0 (11.9) | 76.1 (12.2) |  | | 78.4 (12.9) | 78.8 (12.8) | |  | 79.0 (11.3) | 79.1 (13.5) | |  |
| HLQ D6 (0-100)^m^ |  | | 2.30 (−0.37 to 4.97) | | | .091 | 1.69 (−0.83 to 4.20) | | .19 | | 1.74 (−0.92 to 4.41) | | | .20 | −0.25 (−2.80 to 2.29) | | | .85 |
|  | 74.4 (14.7) | 76.3 (15.1) | 73.5 (15.6) | 76.9 (14.9) | |  | 74.0 (15.5) | 76.8 (13.4) |  | | 75.9 (14.6) | 78.7 (14.7) | |  | 76.3 (13.8) | 77.2 (14.3) | |  |
| HLQ D7 (0-100)^m^ |  | | 1.01 (−1.42 to 3.44) | | | .42 | 1.44 (−1.11 to 3.99) | | .27 | | 2.12 (−0.43 to 4.68) | | | .10 | 0.57 (−2.12 to 3.25) | | | .68 |
|  | 71.0 (14.6) | 72.1 (14.9) | 71.7 (14.3) | 73.4 (14.7) | |  | 71.3 (15.1) | 73.4 (14.1) |  | | 73.1 (13.6) | 75.8 (14.1) | |  | 73.7 (14.7) | 74.9 (14.3) | |  |
| HLQ D8 (0-100)^m^ |  | | 0.26 (−1.84 to 2.36) | | | .81 | 0.35 (−1.91 to 2.60) | | .76 | | 1.28 (−1.06 to 3.61) | | | .28 | −1.40 (−3.62 to 0.83) | | | .22 |
|  | 79.6 (11.8) | 81.3 (11.4) | 79.9 (11.4) | 81.1 (13.2) | |  | 79.7 (12.1) | 81.0 (11.0) |  | | 79.9 (11.6) | 82.1 (12.0) | |  | 82.2 (10.8) | 81.8 (12.5) | |  |
| HLQ D9 (0-100)^m^ |  | | 0.51 (−1.67 to 2.69) | | | .65 | 0.01 (−2.14 to 2.17) | | .99 | | 1.38 (−0.99 to 3.75) | | | .25 | −0.51 (−2.72 to 1.71) | | | .65 |
|  | 84.0 (10.7) | 85.4 (11.3) | 84.5 (11.3) | 85.7 (12.1) | |  | 84.7 (12.1) | 85.5 (10.9) |  | | 85.0 (10.6) | 87.1 (11.7) | |  | 86.4 (11.3) | 86.6 (11.7) | |  |

^a^ n=141 for treatment choices (observed); n=226 for all others.

^b^ n=116 for treatment choices (observed); n=214 for all others.

^c^ MBP: MyBackPain.

^d^ n=197 for RMDQ; n=172 for AQoL; n=180 for pain VAS; n=171 for treatment choices (stated); n=83 for treatment choices (observed); n=185 for all others.

^e^ n=167 for RMDQ; n=156 for AQoL; n=155 for pain VAS; n=156 for treatment choices (stated); n=63 for treatment choices (observed); n=161 for all others.

^f^ n=175 for RMDQ; n=153 for AQoL; n=154 for pain VAS; n=153 for treatment choices (stated); n=73 for treatment choices (observed); n=167 for all others.

^g^ n=158 for RMDQ; n=150 for AQoL; n=140 for pain VAS; n=149 for treatment choices (stated); n=83 for treatment choices (observed); n=154 for all others.

^h^ n=163 for RMDQ; n=140 for AQoL; n=145 for pain VAS; n=140 for treatment choices (stated); n=67 for treatment choices (observed); n=148 for all others.

^i^ n=145 for RMDQ; n=131 for AQoL; n=131 for pain VAS; n=131 for treatment choices (stated); n=67 for treatment choices (observed); n=138 for all others.

^j^ n=161 for RMDQ; n=147 for AQoL; n=153 for pain VAS; n=148 for treatment choices (stated); n=89 for treatment choices (observed); n=152 for all others.

^k^ n=148 for RMDQ; n=140 for AQoL; n=143 for pain VAS; n=140 for treatment choices (stated); n=72 for treatment choices (observed); n=146 for all others.

^l^ HLQ Dx: Health Literacy Domain Dimension × (converted to a 100-point scale).

^m^For conversion of HLQ data (0-100 scale) to conventional 1-4 scale = value*3/100+1.

^n^ RMDQ: Roland Morris Disability Questionnaire.

^o^ VAS: visual analogue scale.
