# Supplementary material for: Effect of a Consumer-Focused Website for Low Back Pain on Health Literacy, Treatment Choices, and Clinical Outcomes: Randomized Controlled Trial
Source: J Med Internet Res. 2021 Jun 15;23(6):e27860. doi: 10.2196/27860 (PMC8277358; doi:10.2196/27860)
Supplement: Multimedia Appendix 4 [file jmir_v23i6e27860_app4.docx]

**Multimedia Appendix 4** Complete case data: Mean (SD) of continuous outcomes and mean difference between groups over time (MyBackPain - Control)

| **Outcome** | **Baseline** | | **1 Month** | | | | **3 Months** | | | | **6 Months** | | | | **12 Months** | | | |
| --- | --- | --- | --- | --- | --- | --- | --- | --- | --- | --- | --- | --- | --- | --- | --- | --- | --- | --- |
|  | **Control ^a^ (n=226)** | **MyBackPain ^b^ (n=214)** | **Mean difference (95%CI)** | | | **p-value** | **Mean difference (95%CI)** | | **p-value** | **Mean difference (95%CI)** | | | | **p-value** | **Mean difference (95%CI)** | | | **p-value** |
|  |  |  | **Control^c^** | | **MBP^d^** |  | **Control^e^** | **MBP^f^** |  | **Control^g^** | | | **MBP^h^** |  | **Control^i^** | | **MBP^j^** |  |
| **Primary outcomes** | | | | | | | | | | | | | | | | | | |
| HLQ D2 |  | | 0.12 (-2.06, 2.29) | | | 0.91 | -0.94 (-3.17, 1.30) | | 0.41 | | 0.84 (-1.48, 3.17) | | | 0.48 | 1.10 (-1.19, 3.39) | | | 0.35 |
|  | 70.8 (14.0) | 71.7 (14.0) | 71.9 (13.2) | 72.9 (13.8) | |  | 73.2 (12.7) | 73.1 (13.6) |  | | 74.0 (13.5) | 75.6 (13.3) | |  | 74.2 (13.7) | 75.8 (14.5) | |  |
| HLQ D3 |  | | 0.26 (-1.64, 2.16) | | | 0.79 | -0.95 (-2.91, 1.02) | | 0.34 | | 1.01 (-1.04, 3.06) | | | 0.33 | 1.17 (-0.84, 3.19) | | | 0.25 |
|  | 74.3 (13.7) | 74.6 (14.5) | 74.2 (12.7) | 74.7 (13.2) | |  | 75.1 (12.6) | 75.1 (12.7) |  | | 74.8 (13.3) | 76.8 (12.3) | |  | 76.3 (11.7) | 78.4 (12.7) | |  |
| **Secondary outcomes** | | | | | | | | | | | | | | | | | | |
| Treatment Choices (Stated) |  | | 0.91 (0.16, 1.67) | | | 0.018 | 0.58 (-0.20, 1.36) | | 0.14 | | 0.83 (0.03, 1.64) | | | 0.043 | 0.73 (-0.06, 1.52) | | | 0.070 |
|  | 5.3 (4.2) | 5.2 (4.6) | 5.1 (4.1) | 6.0 (4.8) | |  | 5.4 (4.6) | 5.7 (4.7) |  | | 5.0 (4.2) | 5.9 (4.7) | |  | 5.2 (4.2) | 5.7 (4.5) | |  |
| Treatment Choices (Observed) |  | | -0.13 (-1.10, 0.85) | | | 0.80 | 0.29 (-0.84, 1.42) | | 0.62 | | -1.08 (-2.07, -0.08) | | | 0.034 | 0.26 (-0.67, 1.19) | | | 0.58 |
|  | 3.1 (3.1) | 2.2 (2.7) | 1.9 (2.6) | 1.6 (2.2) | |  | 2.0 (2.8) | 1.6 (2.9) |  | | 3.1 (2.5) | 2.1 (2.4) | |  | 2.2 (2.6) | 2.2 (1.8) | |  |
| RMDQ |  | | 0.40 (-0.55, 1.34) | | | 0.41 | 0.53 (-0.44, 1.51) | | 0.28 | | -0.09 (-1.10, 0.91) | | | 0.86 | 0.06 (-0.95, 1.06) | | | 0.91 |
|  | 9.3 (5.7) | 8.5 (6.0) | 7.0 (6.0) | 7.0 (5.8) | |  | 7.1 (6.2) | 7.0 (6.0) |  | | 7.2 (6.5) | 6.5 (6.3) | |  | 7.1 (6.3) | 6.5 (6.2) | |  |
| Quality of Life (utility score) |  | | 0.00 (-0.02, 0.03) | | | 0.70 | 0.00 (-0.02, 0.03) | | 0.68 | | 0.02 (-0.01, 0.04) | | | 0.14 | 0.01 (-0.02, 0.03) | | | 0.56 |
|  | 0.54 (0.20) | 0.55 (0.23) | 0.55 (0.21) | 0.57 (0.22) | |  | 0.56 (0.21) | 0.58 (0.23) |  | | 0.54 (0.22) | 0.57 (0.23) | |  | 0.56 (0.22) | 0.58 (0.23) | |  |
| Pain VAS |  | | 0.12 (-4.38, 4.62) | | | 0.96 | -0.40 (-5.13, 4.32) | | 0.87 | | -0.19 (-5.03, 4.66) | | | 0.94 | -0.63 (-5.35, 4.08) | | | 0.79 |
|  | 54.2 (18.2) | 51.8 (18.8) | 48.6 (23.5) | 47.4 (25.2) | |  | 46.9 (24.4) | 46.0 (24.7) |  | | 45.8 (24.7) | 45.8 (26.3) | |  | 48.1 (25.7) | 46.6 (26.4) | |  |
| HLQ D1 |  | | 0.50 (-1.80, 2.81) | | | 0.67 | 0.20 (-2.18, 2.57) | | 0.87 | | 2.02 (-0.45, 4.50) | | | 0.11 | 1.63 (-0.81, 4.07) | | | 0.19 |
|  | 78.1 (15.2) | 78.0 (15.6) | 78.1 (14.6) | 78.5 (15.7) | |  | 77.6 (15.3) | 77.9 (14.3) |  | | 78.0 (15.0) | 79.8 (13.9) | |  | 79.1 (14.8) | 79.5 (14.6) | |  |
| HLQ D4 |  | | -0.08 (-2.20, 2.05) | | | 0.94 | -1.33 (-3.52, 0.85) | | 0.23 | | -0.29 (-2.57, 1.99) | | | 0.80 | -0.64 (-2.89, 1.61) | | | 0.58 |
|  | 69.4 (14.9) | 67.0 (15.2) | 69.5 (14.9) | 68.0 (15.8) | |  | 71.1 (14.2) | 68.1 (13.6) |  | | 72.1 (16.3) | 69.9 (14.9) | |  | 73.1 (15.0) | 70.2 (15.7) | |  |
| HLQ D5 |  | | 0.63 (-1.21, 2.47) | | | 0.50 | -1.30 (-3.20, 0.59) | | 0.18 | | 0.17 (-1.80, 2.15) | | | 0.86 | 0.51 (-1.44, 2.46) | | | 0.61 |
|  | 76.3 (12.5) | 77.1 (11.4) | 75.4 (11.1) | 76.8 (12.8) | |  | 77.2 (11.7) | 76.1 (11.7) |  | | 78.5 (12.4) | 78.7 (12.0) | |  | 79.1 (10.6) | 79.7 (12.8) | |  |
| HLQ D6 |  | | 1.42 (-0.70, 3.54) | | | 0.19 | 1.05 (-1.13, 3.22) | | 0.35 | | 1.28 (-0.98, 3.54) | | | 0.27 | -0.66 (-2.89, 1.57) | | | 0.56 |
|  | 74.4 (14.7) | 76.3 (15.1) | 73.8 (15.4) | 77.0 (15.2) | |  | 73.8 (15.7) | 77.2 (13.2) |  | | 75.9 (14.5) | 78.8 (14.4) | |  | 76.6 (13.8) | 77.1 (14.4) | |  |
| HLQ D7 |  | | -0.06 (-2.08, 1.96) | | | 0.95 | 0.30 (-1.77, 2.38) | | 0.77 | | 1.11 (-1.04, 3.27) | | | 0.31 | 0.31 (-1.81, 2.44) | | | 0.77 |
|  | 71.0 (14.6) | 72.1 (14.9) | 72.1 (14.2) | 73.4 (15.0) | |  | 71.1 (15.2) | 73.5 (14.2) |  | | 73.2 (13.6) | 75.9 (13.8) | |  | 73.9 (15.0) | 75.4 (14.0) | |  |
| HLQ D8 |  | | -0.15 (-2.02, 1.71) | | | 0.87 | 0.21 (-1.71, 2.12) | | 0.83 | | 1.35 (-0.64, 3.35) | | | 0.18 | -0.75 (-2.72, 1.21) | | | 0.45 |
|  | 79.6 (11.8) | 81.3 (11.4) | 79.8 (11.5) | 81.2 (13.5) | |  | 79.8 (12.0) | 81.3 (10.9) |  | | 80.3 (11.5) | 82.8 (11.5) | |  | 82.2 (10.7) | 82.3 (12.4) | |  |
| HLQ D9 |  | | -0.09 (-1.95, 1.77) | | | 0.93 | -0.66 (-2.57, 1.25) | | 0.50 | | 1.02 (-0.96, 3.00) | | | 0.31 | -0.76 (-2.71, 1.19) | | | 0.45 |
|  | 84.0 (10.7) | 85.4 (11.3) | 84.4 (11.3) | 85.7 (12.3) | |  | 85.1 (12.0) | 85.9 (10.6) |  | | 85.4 (10.3) | 87.7 (11.1) | |  | 87.0 (11.1) | 87.0 (11.5) | |  |

MBP – MyBackPain; VAS – visual analogue scale; RMDQ – Roland Morris Disability Questionnaire; HLQ Dx – Health Literacy Domain Dimension x (converted to 100 point scale); For conversion of HLQ data (0-100 scale) to conventional 1-4 scale = value*3/100+1.

^a^ n=141 for treatment choices (observed), n=226 for all others

^b^ n=116 for treatment choices (observed), n=214 for all others

^c^ n=197 for RMDQ, n=172 for AQoL, n=180 for Pain VAS, n=171 for treatment choices (stated), n=83 for treatment choices (observed), n=185 for all others

^d^ n=167 for RMDQ, n=156 for AQoL score, n=155 for Pain VAS, n=156 for treatment choices (stated), n=63 for treatment choices (observed), n=161 for all others

^e^ n=175 for RMDQ, n=153 for AQoL, n=154 for Pain VAS, n=153 for treatment choices (stated), n=73 for treatment choices (observed), n=167 for all others

^f^ n=158 for RMDQ, n=150 for AQoL, n=140 for Pain VAS, n=149 for treatment choices (stated), n=83 for treatment choices (observed), n=154 for all others

^g^ n=163 for RMDQ, n=140 for AQoL, n=145 for Pain VAS, n=140 for treatment choices (stated), n=67 for treatment choices (observed), n=148 for all others

^h^ n=145 for RMDQ, n=131 for AQoL, n=131 for Pain VAS, n=131 for treatment choices (stated), n=67 for treatment choices (observed), n=138 for all others

^i^ n=161 for RMDQ, n=147 for AQoL, n=153 for Pain VAS, n=148 for treatment choices (stated), n=89 for treatment choices (observed), n=152 for all others

^j^ n=148 for RMDQ, n=140 for AQoL, n=143 for Pain VAS, n=140 for treatment choices (stated), n=72 for treatment choices (observed), n=146 for all other
