# Supplementary material for: Effect of a Consumer-Focused Website for Low Back Pain on Health Literacy, Treatment Choices, and Clinical Outcomes: Randomized Controlled Trial
Source: J Med Internet Res. 2021 Jun 15;23(6):e27860. doi: 10.2196/27860 (PMC8277358; doi:10.2196/27860)
Supplement: Multimedia Appendix 5 [file jmir_v23i6e27860_app5.docx]

**Multimedia Appendix 5** Complete case data: Number (percentage) of participants choosing treatment types (the binary outcomes).

| **Month** | **Control** | **Number Missing** | **MyBackPain** | **Number Missing** | **Risk ratio (95% CI)** | **p-value** |  |  |
| --- | --- | --- | --- | --- | --- | --- | --- | --- |

| **Observed treatment choice harmful/no effect** | | | | | | | | |
| --- | --- | --- | --- | --- | --- | --- | --- | --- |
| **1** | 20/83 (24.10) | 143 | 17/63 (26.98) | 151 | 1.34 (0.40, 2.28) | 0.48 |  |  |
| **3** | 12/73 (16.44) | 153 | 13/52 (25.00) | 162 | 2.47 (0.15, 4.80) | 0.21 |  |  |
| **6** | 11/67 (16.42) | 159 | 11/67 (16.42) | 147 | 1.65 (0.29, 3.02) | 0.35 |  |  |
| **12** | 18/89 (20.22) | 137 | 10/72 (13.89) | 142 | 1.32 (0.30, 2.33) | 0.54 |  |  |
| **Observed treatment choice recommended** | | | | | | | | |
| **1** | 55/83 (66.27) | 143 | 40/63 (63.49) | 151 | 1.02 (0.76, 1.28) | 0.87 |  |  |
| **3** | 49/73 (67.12) | 153 | 37/52 (71.15) | 162 | 1.29 (1.01, 1.58) | 0.045 |  |  |
| **6** | 54/67 (80.60) | 159 | 47/67 (70.15) | 147 | 0.85 (0.66, 1.03) | 0.11 |  |  |
| **12** | 65/89 (73.03) | 137 | 54/72 (75.00) | 142 | 0.96 (0.76, 1.15) | 0.65 |  |  |
