# Supplementary material for: Effect of a Consumer-Focused Website for Low Back Pain on Health Literacy, Treatment Choices, and Clinical Outcomes: Randomized Controlled Trial
Source: J Med Internet Res. 2021 Jun 15;23(6):e27860. doi: 10.2196/27860 (PMC8277358; doi:10.2196/27860)
Supplement: Multimedia Appendix 6 [file jmir_v23i6e27860_app6.docx]

**Multimedia Appendix 6** Evidence for effectiveness standards

| **Evidence category** | **Evidence standard** | **Evidence of best practice standard** |
| --- | --- | --- |
| Credibility with health care professionals | Viewed as useful and relevant by professional experts and involved in design | Published evidence of involvement of experts in design/content development [43] and review [21] |
| Relevance to current care pathways | Evidence of implementation | Published evidence implementation in collaboration with Arthritis Australia [21] |
| Acceptability with users | Evidence of involvement of users in design | Published evidence [10, 21]; Qualitative study of user experience [32] |
| Equalities considerations | Improve access to care in hard-to-reach populations |  |
| Accurate and reliable measurements | Accurate, reproducible and relevant data – guidance tools | Treatment tailoring based on validated Pick-up tool (Acute)[25] and StartBack (Chronic)[44]. |
| Reliable information content | Valid aligned to best available sources, accurate, up to date, reviewed by relevant experts, comprehensive | Published evidence of “key messages”; Involvement of international expert panel in treatment comparison summaries [21] |
| Ongoing data collection to show usage of the DHT | Commitment to ongoing data collection | Ongoing use data collection (Arthritis Australia) |
| Ongoing data collection to show value of the DHT. | Commitment to ongoing data collection to show user outcomes (if relevant) or user satisfaction | Ongoing qualitative research e.g., [32]; |
| Quality and safeguarding | Safeguarding measures for peer-support and other communication functions | Not yet applicable |

Adapted from: Evidence of effectiveness standards for “Tier 2 digital health technology (DHT) - DHTs which help users to understand healthy living and illnesses” from NICE Evidence Standards Framework for Digital Health Technologies [41].
